# High-normal blood pressure and long-term risk of type 2 diabetes: 35-year prospective population based cohort study of men

**DOI:** 10.1186/1471-2261-12-89

**Published:** 2012-10-15

**Authors:** Christina Hedén Stahl, Masuma Novak, Georgios Lappas, Lars Wilhelmsen, Lena Björck, Per-Olof Hansson, Annika Rosengren

**Affiliations:** 1 Department of Molecular and Clinical Medicine, the Sahlgrenska Academy, University of Gothenburg, Gothenburg SE-416 85, Sweden; 2 Preventive Cardiology, the Sahlgrenska Academy, University of Gothenburg, Gothenburg, Sweden

**Keywords:** Incidence, Type 2 diabetes, High-normal blood pressure, Prehypertension, Cohort study, Epidemiology, Sweden

## Abstract

**Background:**

The link between type 2 diabetes and hypertension is well established and the conditions often coexist. High normal blood pressure, defined by WHO-ISH as systolic blood pressure (SBP) 130–139 mm Hg or diastolic blood pressure (DBP) 85–89 mm Hg, has been found to be an independent predictor for type 2 diabetes in studies, although with relatively limited follow-up periods of approximately 10 years. The aim of this study was to investigate whether hypertension, including mildly elevated blood pressure within the normal range, predicted subsequent development of type 2 diabetes in men over an extended follow-up of 35 years.

**Methods:**

Data were derived from the Gothenburg Primary Prevention Study where a random sample of 7 494 men aged 47–55 years underwent a baseline screening investigation in the period 1970–1973. A total of 7 333 men were free from previous history of diabetes at baseline. During a 35-year follow-up diabetes was identified through the Swedish hospital discharge and death registries. The cumulative risk of diabetes adjusted for age and competing risk of death was calculated. Using Cox proportional hazard models we calculated the multiple adjusted hazard ratios (HR) (95% confidence interval (CI)) for diabetes at different blood pressure levels.

**Results:**

During a 35-year follow-up, 956 men (13%) were identified with diabetes. The 35-year cumulative risk of diabetes after adjusting for age and competing risk of death in men with SBP levels <130 mm Hg, 130–139 mm Hg, 140–159 mm Hg and ≥160 mm Hg were 19%, 30%, 31% and 49%, respectively. The HR for diabetes adjusted for age, body mass index (BMI), cholesterol, antihypertensive treatment, smoking, physical activity and occupation were 1.43 (95% CI 1.12-1.84), 1.43 (95% CI 1.14-1.79) and 1.95 (95% CI 1.55-2.46) for men with SBP 130–139 mm Hg, 140–159 mm Hg, and ≥ 160 mm Hg, respectively (reference; SBP<130 mm Hg).

**Conclusion:**

In this population, at mid-life, even high-normal SBP levels were shown to be a significant predictor of type 2 diabetes, independently of BMI and other conventional type 2 diabetes risk factors over an extended follow-up.

## Background

The incidence of type 2 diabetes is increasing rapidly worldwide, due mainly to an aging population, rapid increases in overweight and obesity, and population growth [1-4]. Diabetes plays a significant role in cardiovascular morbidity and mortality, and therefore it is important to identify individuals at increased risk of developing diabetes in order to introduce preventive strategies [5]. Patients with type 2 diabetes often have hypertension and other cardiovascular risk factors, increasing the risk of cardiovascular morbidity still further [6]. Previous studies have shown that hypertension is an independent predictor of type 2 diabetes [7,8]. In recent years, studies have indicated that blood pressure in the upper normal range also predicts incident diabetes [9-13]. If so, even persons with mildly raised blood pressure might benefit from increased surveillance of glucose levels and preventive strategies against type 2 diabetes. The follow-up time in studies examining the relationship between high normal blood pressure and diabetes is often 10 years or less, whereas most cardiovascular disease (CVD) events occur late in life. A risk factor indicating an increased risk of an outcome after 10 years is not necessarily of importance in a longer follow-up [14,15]. For example, we have shown that smoking, a strong risk factor in midlife for coronary heart disease (CHD), becomes less important over time and was no longer a significant risk factor after 21 years [14]. Although we know from the Framingham study that risk factors in midlife are important for long-term outcomes and the lifetime risk of cardiovascular disease, this has not been well studied for diabetes. To our knowledge, this is the first study to examine whether the association between midlife blood pressure and incidence of type 2 diabetes persists in the general population over an extended follow-up until old age.

## Methods

### Study population

Data were derived from the intervention group of the multifactor Primary Prevention Study, which began in 1970. The study population and design have been described in detail elsewhere [16]. In brief, all men (n= approximately 30 000) living in the city of Gothenburg, Sweden and born between 1915 and 1925 (except those born in 1923) were randomised into three equally large groups, where the men in one of the groups (i.e. the intervention group, n=10 004) were offered a medical examination to identify and treat risk factors, with the remaining men randomised into two control groups (i.e. groups without any interventions) [16]. The intervention criteria in the study were antihypertensive treatment if systolic blood pressure (SBP) exceeded 175 mm Hg or diastolic blood pressure (DBP) 115 mm Hg, dietary advice if serum cholesterol levels were above 260 mg per 100 ml (=6.8 mmol-1) and referral to anti-smoking clinics for participants who smoked 15 cigarettes or more per day. Treatment was offered at specialist clinics. During the first 12-year follow-up, there were no significant differences in outcomes with respect to cardiovascular disease or all-cause mortality between the intervention and control groups [16]. Thus, despite the fact that the men took part in an intervention study, we consider the study cohort to be representative of the general Gothenburg male population. All participants gave their informed consent to participate in the study, which was approved by the Ethics Committee for Medical Research at the University of Gothenburg.

A total of 7 494 men (75% of the sample) took part in the baseline screening examination which took place between January 1970 and March 1973. All participants completed a postal questionnaire before the examination. Of the men who took part in the baseline screening, 149 men had a prior history of diabetes and were excluded from the analyses. Prior history of diabetes at baseline was based on participants’ self-reports of diagnosed diabetes indicated by the answer ‘yes’ to the question ‘Has a physician ever told you that you have diabetes?’ Participants with missing information on SBP were also excluded from the analysis (12 cases). The remaining 7 333 men constitute the basis of the present study and were followed up for a maximum period of 35 years.

### Measurements and definitions

For the purpose of the present study, information selected from the baseline postal questionnaire included smoking habits, leisure time physical activity, antihypertensive treatment (yes/no) and occupation. Smoking habit was divided into never smokers and former smokers of more than 1 month’s duration or current daily smokers. Physical activity was divided into low (sedentary), moderate and regular exercise. Occupation was classified according to the Swedish socioeconomic classification system (SEI) and defined as either manual (SEI 1–3 or unclassified) or non-manual (SEI 4–5) [17].

The baseline screening examinations were performed in the afternoon between 4.00 pm and 7.00 pm after a working day. Weight was measured to the nearest 0.10 kg and height to the nearest 0.01 metre. Body mass index (BMI) (weight in kilograms divided by measured height in square metres) was categorised as <25 (normal), 25–30 (overweight) and >30 kg/m^2^ (obese). Serum cholesterol concentration was determined according to standard laboratory procedures. Blood pressure was taken from the right arm with the participant seated, after a 4–5 minute rest and by physicians trained to carry out the process repeatedly in a similar manner. A mercury manometer was used and measured to the nearest 2 mm Hg. A large proportion of the participants were found to have high blood pressure. In a random subsample (n=84/2180) blood pressure was also measured in the morning two weeks later. Mean SBP was then 7.6 mm Hg lower and mean DBP 8.9 mm Hg lower in comparison to the screening blood pressure. Among those with the highest blood pressure levels during the screening, the mean SBP and DBP was even lower two weeks later; 16.1 mm Hg and 18.0 mm Hg respectively. The conclusion made by the original investigators of the Primary Prevention Study was that the circumstances of the blood pressure measurements probably influenced the values and that there was no reason to believe that blood pressure levels were substantially higher in Gothenburg than in other populations at that time [18]. Missing data on all covariates was 1.3% or below.

### Ascertainment of diabetes incidence

All participants were followed from the date of their baseline examination until 31 December 2008 using their unique personal identification number. A computer file of the study cohort was run against the Swedish national register on cause of death and the Swedish hospital discharge register. This procedure was reviewed and approved by the Ethics Committee.

The hospital discharge register has operated on a nationwide basis since 1987, but all discharges from Gothenburg hospitals have been entered in the national register since 1970 (except 1976 owing to a legislative change for that single year). Type 2 diabetes was identified from the hospital discharge as a primary or a secondary diagnosis using a code of 250 (International Classification of Diseases, Eighth revision [ICD 8], 250 (International Classification of Diseases, Ninth revision [ICD 9]) or E10-E14 (International Classification of Diseases, 10th revision [ICD 10]). A death certificate diagnosis of diabetes as an underlying cause by any of the diagnostic codes above in a man with no prior diabetes diagnosis was also accepted. Of the men, only a very small minority 5.8% (428/7333) had never been hospitalised before the end of the follow-up. At 31 December 2008, 98.2% (7 198/7333) of the participants had a registration in either the Swedish national register of cause of death or the Swedish hospital discharge register.

### Classification of blood pressure levels

The blood pressure categories are based on the WHO-ISH definition [19] where SBP is classified into following four categories: < 130 (normal), 130–139 (high-normal), 140–159 (mild hypertension) and ≥160 (moderate and severe hypertension) mm Hg; and DBP into following three categories: <85 (normal), 85–89 (high-normal), ≥90 (hypertension) mm Hg. We regarded SBP and DBP as independent risk factors and analysed them separately.

### Statistics

Descriptive statistics are presented in terms of frequencies and percentages for categorical variables and in terms of mean with standard deviation for continuous variables. Differences in the distribution of baseline characteristics across the blood pressure categories were examined by chi-square trend test for categorical variables and by Spearman correlation test for continuous variables. All *p*-values are 2-sided and values <0.05 are considered statistically significant. We calculated age-adjusted diabetes incidence rates per 100 000 person years for each blood pressure category. Time at risk was calculated from the baseline examination between January 1970 and March 1973 to first hospitalisation with a diagnosis of diabetes (as a principal or a secondary diagnosis), to death or to 31 December 2008. Cox proportional hazard models were used to analyse time at risk and the association with blood pressure levels. In the blood pressure categories the lowest blood pressure group was used as the reference. Estimates from the Cox proportional hazard models are presented as hazard ratio (HR) and 95% confidence intervals (95% CI). Three models were used; first we computed the age adjusted HR, then the age and BMI adjusted HR and finally the multiple adjusted HR where we adjusted for age, BMI, cholesterol level, use of antihypertensive treatment, smoking, physical activity and occupational class. The assumption of the proportional hazard was tested and holds for our model. In order to investigate the possible impact of residual confounding, we have performed sensitivity analyses using Cox proportional hazard models and calculated the age and multiple adjusted hazard ratios of diabetes for different blood pressure categories in different BMI and smoking groups. Due to the long follow-up and the age of the participants at study entry, a large proportion, 75% (5644/7494), had died by the end of the study. In order to take into account death from other causes we present curves based on the cumulative risk of diabetes adjusted for age and competing risk of death over the follow-up period according to levels of blood pressure at baseline. All analyses were performed using SAS software version 9.2 (SAS institute, Cary, NC, USA) and Statistical package R2.15 version.

## Results

The median follow-up time in our study was 28 years. The mean age of the study subjects at baseline was 51.6 years (standard deviation (SD) 2.3), the mean SBP was 149 (SD 22) and the mean DBP was 95 (SD 13) mm Hg. After 10 years of follow-up, 1.3% (96/7333) of the men were discharged from hospital with a principal or secondary diagnosis of diabetes or were diagnosed as having diabetes on their death certificate. After 35 years of follow-up the proportion, without adjustment for competing risk of death, was 13.0% (956/7333) and the crude incidence of diabetes was 509 per 100 000 person years. Of the 956 diabetes cases, 54 were from death certificates and thus 902 from hospital discharge registers.

Baseline characteristics for participants in the different SBP groups are shown in Table 1. The proportion of men in the SBP groups <130 mm Hg, 130–139 mm Hg, 140–159 mm Hg, and ≥160 mm Hg were 17%, 18%, 36%, and 29% respectively. Participants in the higher SBP groups were slightly older, had higher BMI and cholesterol levels, were more likely to use antihypertensive medication, less likely to be physically active, current smokers or from the non-manual occupational class than participants in the lower blood pressure groups.

**Table 1:** Baseline characteristics according to systolic blood pressure categories

		**Systolic blood pressure categories**				
	**All**	**<130 mm Hg**	**130-139 mm Hg**	**140-159 mm Hg**	**≥160 mm Hg**	
**Characteristics**	**N = 7 333**	**(n = 1278)**	**(n = 1315)**	**(n = 2623)**	**(n = 2117)**	p-values*
Age, years, mean (SD)	51.6	51.2	51.3	51.6	51.9	<0.0001
	(2.3)	(2.3)	(2.4)	(2.3)	(2.1)	
Body Mass Index kg/m^2^, mean (SD)	25.5	24.4	25.2	25.6	26.3	<0.0001
	(3.2)	(2.9)	(2.9)	(3.2)	(3.5)	
Obesity. BMI ≥30, % (n)	8.1	3.4	5.6	8.2	12.6	<0.0001
	(597)	(43)	(73)	(214)	(267)	
Diastolic blood pressure, mm Hg, mean (SD)	95	82	88	94	107	<0.0001
	(13)	(8)	(8)	(8)	(12)	
Hypertension treatment, % (n)	5.4	0.7	0.7	3.2	13.8	<0.0001
	(396)	(9)	(9)	(85)	(293)	
Serum cholesterol mmol/L, mean (SD)	6.46	6.22	6.47	6.47	6.61	<0.0001
	(1.15)	(1.08)	(1.10)	(1.17)	(1.18)	
Never smokers, % (n)	29.5	27.2	27.1	29.7	32.0	0.0004
	(2152)	(347)	(355)	(775)	(675	
Former smokers, % (n)	20.4	19.0	20.9	21.3	20.0	0.68
	(1493)	(242)	(273)	(555)	(423)	
Current smokers, % (n)	50.1	53.8	52.0	49.0	48.0	0.0004
	(3660)	(686)	(681)	(1279)	(1014)	
Physically active, % (n)	16.0	18.3	17.6	16.1	13.5	<0.0001
	(1156)	(232)	(228)	(414)	(282)	
Non-manual occupation	27.9	31.2	29.4	27.8	25.1	<0.0001
	(2044)	(399)	(386)	(728)	(531)	

SD = standard deviation. BMI=Body Mass Index. *P-values calculated by chi-square trend test for categorical variables and by Spearman correlation test for continuous variables.

Table 2 presents age, BMI and multivariable adjusted HRs for diabetes by different blood pressure categories. The highest risk was in the highest SBP category where multivariable adjusted HR for incident diabetes was 1.95 (95% CI 1.55-2.46) in men with SBP ≥160 mm Hg compared to SBP <130 mm Hg (referent). The HRs were also significantly higher in the two lower SBP categories, including the high-normal category of 130 to 139 mm Hg, and to approximately the same extent. The inclusion of BMI reduced the estimates significantly more than any other variable, taken separately or together. The multiple adjusted HR for diabetes was 1.34 (95% CI 1.12-1.62) in men with DBP ≥90 mm Hg compared to DBP below 85 mm Hg (referent). In the DBP category 85 to 89 mm Hg there was no significantly increased risk compared to DBP below 85 mm Hg.

**Table 2:** Hazard ratios for diabetes incidence by blood pressure categories

**Blood pressure categories**	**Number at risk**	**Diabetes cases**	**Person years**	**Diabetes cases per 100 000 person years**	**Age adjusted hazard ratios (95% CI)**	**Age and BMI adjusted hazard ratios (95% CI)**	Age and multivariable adjusted* hazard ratios (95% CI)
**Systolic blood pressure**							
< 130 mm Hg (normal)	1 279	109	36292	300	ref.	ref.	ref.
130 – 139 mm Hg (high-normal)	1 315	159	35541	447	1.56 (1.22-1.99)	1.39 (1.09-1.78)	1.43 (1.12-1.84)
140 – 159 mm Hg (mild hypertension)	2622	330	69845	472	1.66 (1.34-2.07)	1.40 (1.13-1.75)	1.43 (1.14-1.79)
≥ 160 mm Hg (moderate/severe)	2117	358	51695	693	2.68 (2.16-3.32)	2.03 (1.63-2.52)	1.95 (1.55-2.46)
Increase per 10 mm Hg					1.16 (1.13-1.18)	1.12 (1.08-1.14)	1.10 (1.07-1.14)
**Diastolic blood pressure**							
< 85 mm Hg (normal)	1628	157	45528	345	ref.	ref.	ref.
85 – 89 mm Hg (high normal)	896	83	24227	343	1.02 (0.78-1.33)	0.95 (0.72-1.23)	0.93 (0.70-1.22)
≥ 90 mm Hg (hypertension)	4809	716	123617	579	1.82 (1.53-2.16)	1.41 (1.18-1.68)	1.34 (1.12-1.62)
Increase per 5 mm Hg					1.14 (1.12-1.17)	1.09 (1.06-1.11)	1.08 (1.06-1.11)

*Multivariable adjusted model included age, body mass index, cholesterol level, antihypertensive treatment, smoking, physical activity and occupational class.

We have also calculated the multivariable adjusted HRs for diabetes by SBP categories in different BMI and smoking categories. The effect of increasing blood pressure on risk of diabetes was similar irrespective of BMI category or smoking status, with no suggestion of an interaction effect (Additional file 1: Table S3).

**Figure 1:**
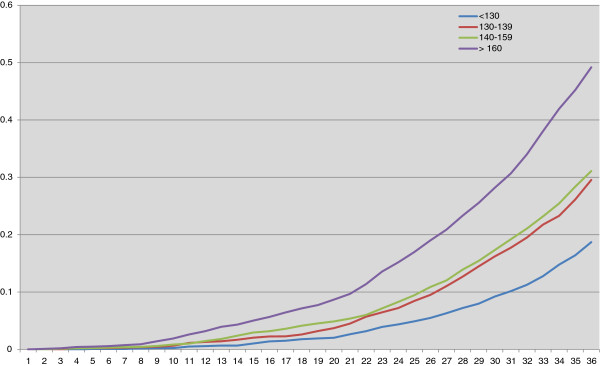
**Cumulative risk of diabetes by different systolic blood pressure categories.** Cumulative risk of diabetes adjusted for age and competing risk of death over the 35-year follow-up based on the level of SBP at baseline (SBP levels: <130 mm Hg, 130–139 mm Hg, 140–159 mm Hg and ≥160 mm Hg).

Figure 1 presents the cumulative risk of diabetes over time adjusted for age and competing risk of death in men in different SBP categories. The 35-year risk of diabetes in the SBP groups <130 mm Hg, 130–139 mm Hg, 140–159 mm Hg and ≥160 mm Hg was considerably higher than without this adjustment, or 19%, 30%, 31% and 49% respectively.

## Discussion

In this prospective study of middle-aged men who were followed for 35 years or until death, we found that even moderately increased SBP predicted the subsequent development of diabetes. The association with SBP was graded, and persisted after adjustment for a variety of potential confounders, with the greatest risk of subsequent development of diabetes in the highest blood pressure group. The finding that blood pressure within the higher normal blood pressure range increases the risk of diabetes is consistent with the findings from earlier studies [9-13], with the added information that the increased risk remains through an extended follow-up until old age. Our median follow-up was 28 years which is considerably longer than in previous studies which were 8.9 [9], 7.8 [10], 10.2 [11], 12.5 [12] or a mean follow-up of 8.3 ±1.0 [13] years. Findings for DBP were similar, albeit less pronounced, with only DBP above 90 mm Hg indicating a later risk of diabetes. This is consistent with some studies [20] which did not find DBP to be a risk factor for incident diabetes whereas other studies did [7].

Compared to other studies [9-13] we have a low proportion of diabetes cases during the first 10 years and this is probably explained by the fact that diabetes is often managed in primary care during the first years after diagnosis. We have a detection delay in the study since we did not have access to primary care data and were able to identify diabetes only as a hospital discharge diagnosis.

We found an inverse relation between smoking and blood pressure, probably due to the fact that smokers weigh less, which has also been described elsewhere [21]. Body weight is a strong determinant for diabetes. The increased risk of diabetes in the higher blood pressure categories was strongly attenuated when adjusting for BMI. This is in coherence with what other studies have found [9,13] and indicates that BMI is the factor with the greatest influence on diabetes risk.

At baseline the participants were asked about antihypertensive medication and the analyses were adjusted for this. Data on new antihypertensive treatment during follow-up was not available. This could be a bias since some antihypertensive drugs are known to increase the risk of developing diabetes [22]. Other studies have indicated that the greatest increase in risk of later development of type 2 diabetes in a hypertensive patient is due to hypertension itself [8] and that the increased risk of diabetes remains after adjusting for specific antihypertensive treatments [23]. Therefore it does not seem plausible that medication could explain more than part of the total effect.

The benefits of considering high-normal blood pressures as a predisease have been debated [24]. Whether this should be labeled predisease or not may not be the issue here, however, but what this and other studies [10-13] show is that the risk of developing diabetes is already increased at blood pressure levels below the limits generally used in considering treatment for hypertension. The clinical impact of these findings should be further analysed in appropriate studies.

The pathophysiological mechanisms underlying the association between high blood pressure and type 2 diabetes are not yet completely identified. Hypertension has been shown to induce microvascular changes [25] and these changes may result in a reduced capacity for insulin mediated glucose uptake in the tissue [26]. Impaired microcirculation in tissues may thus be the connecting link between hypertension and insulin resistance [27]. Microcirculation has also been shown to be impaired by obesity; microcirculation has therefore emerged as a potential common denominator for several risk factors for metabolic syndrome [28,29]. Hypertension is also associated with endothelial dysfunction [30] and markers of endothelial dysfunction have in turn been demonstrated to precede the development of diabetes [31]. Another possible linking factor between blood pressure and diabetes is inflammation. Hypertension [32] and diabetes [33] are both known to be associated with an increase in inflammatory markers.

### Strengths and limitations

The strengths of the present study include a large number of unselected participants from the general population, prospective longitudinal design, extended follow-up, and hence the identification of a large number of diabetes cases (956 cases or 13%) compared to other studies [10-13]. Even so, there are also a number of limitations to be considered. Firstly, participants who reported no history of diabetes during the screening examination were considered to be free from diabetes but neither blood glucose analyses nor an oral glucose tolerance test were performed. Therefore some of the participants might have had undiagnosed diabetes at inclusion. However, the majority of diabetes cases were identified at least a decade after the screening, so it is unlikely that the few cases that might have been included could significantly affect our results. We have also performed an analysis in which all the diabetes cases that are identified during the first 7.5 years were excluded which did not alter our results. Secondly, diabetes was defined as a discharge from hospital with a primary or secondary diagnosis code or a death certificate diagnosis of diabetes as an underlying cause. Some of the participants who developed diabetes but did not visit hospital during the follow-up period might not have been identified. Even so, the majority of them did attend hospital at some point (94.2%) and most of them several times, particularly in the last few years of the follow-up. Additionally, in our research group we have carefully followed another cohort of men, the men born in 1913 in Gothenburg, for whom we also have data from primary health care centres as well as diagnosis data from the registers. After 30 years of follow-up 13% of these men had developed type 2 diabetes (unpublished data) which is a similar figure to the proportion of men who developed diabetes in the Primary Prevention Study. Therefore, we believe that most of the diabetes cases were actually detected in the current analyses. Thirdly, the older ICD versions (ICD 8 and ICD 9) do not distinguish between type 1 and type 2 diabetes; however, given the age of the population, few, if any cases would have been type 1 diabetes. Fourthly, a remaining issue could be whether people reacting with high blood pressure at the screening were more prone to develop diabetes. This is something we are unable to adjust for in the study and, as we see it, a separate issue that must be analysed in another study. Fifthly, information on important risk factors other than blood pressure may be considered to be somewhat crude; therefore our results might have been affected by residual confounding. For instance, we have only information about BMI and not hip-waist ratio. One study investigating the relationship between blood pressure and diabetes incidence [13] had both BMI and hip-waist measurement and there was a very minor difference in diabetes risk when using BMI instead of hip-waist ratio. For physical activity, no figures for hours per week are available in our study, just classification into 3 groups (sedentary, moderate and active). In their study, Conen et al. [11] had information regarding the number of hours per week of physical activity, and even after controlling for this, the relationship between blood pressure and diabetes remained. In our study no information on smoking duration is available, and nor do we have dietary information. Diet pattern has been shown to affect diabetes incidence in a previous study [34] but to our knowledge there are no studies concerning the relationship between blood pressure and diabetes that have adjusted for dietary pattern. Nevertheless, it seems unlikely that information about smoking duration and dietary pattern would eliminate the significance in our findings. Moreover, the results from our sensitivity analysis showed that the problem of residual confounding is likely to be negligible. Finally, we have only baseline information on all of the covariates. Many of the covariates are time dependent and fluctuations occur. Therefore we might not have captured the total influence of the covariates on the association between blood pressure levels and diabetes incidence.

## Conclusion

In conclusion, the present study has shown that hypertension and high-normal systolic blood pressure at mid life is a significant risk factor for type 2 diabetes in men over a 35-year follow-up period. The association between blood pressure and type 2 diabetes was independent of BMI and other conventional risk factors. Even so, further studies are needed to assess clinical impact of these findings and whether physicians should consider high-normal blood pressure patients for intense diabetes screening.

## Competing interests

The authors declare that they have no competing interests.

## Authors’ contributions

CH, AR, PH and MN constructed and designed the current project. CH, AR, GL and MN performed the analysis and interpreted the data. CH and MN wrote the manuscript, PH, LW and LB revised the manuscript. LW is the principal investigator of the Primary Prevention Study. All authors approved the final manuscript for publication.

## Pre-publication history

The pre-publication history for this paper can be accessed here:

http://www.biomedcentral.com/1471-2261/12/89/prepub

## Supplementary Material

Additional file 1 **Table S3. a**. Hazard ratio for diabetes by systolic blood pressure (SBP) categories in different BMI categories. **b**; Hazard ratio for diabetes by systolic blood pressure (SBP) categories in different smoking categories. Click here for file

## References

[B1] Zimmet P Alberti KG Shaw J Global and societal implications of the diabetes epidemic Nature 2001 414 6865 782 787 10.1038/414782a 11742409

[B2] Danaei G Finucane MM Lu Y Singh GM Cowan MJ Paciorek CJ Lin JK Farzadfar F Khang YH Stevens GA National, regional, and global trends in fasting plasma glucose and diabetes prevalence since 1980: systematic analysis of health examination surveys and epidemiological studies with 370 country-years and 2.7 million participants Lancet 2011 378 9785 31 40 10.1016/S0140-6736(11)60679-X 21705069

[B3] Tobias M Global control of diabetes: information for action Lancet 2011 378 9785 3 4 10.1016/S0140-6736(11)60604-1 21705073

[B4] Nolan CJ Damm P Prentki M Type 2 diabetes across generations: from pathophysiology to prevention and management Lancet 2011 378 9786 169 181 10.1016/S0140-6736(11)60614-4 21705072

[B5] Sarwar N Gao P Seshasai SR Gobin R Kaptoge S Di Angelantonio E Ingelsson E Lawlor DA Selvin E Stampfer M Diabetes mellitus, fasting blood glucose concentration, and risk of vascular disease: a collaborative meta-analysis of 102 prospective studies Lancet 2010 375 9733 2215 2222 10.1016/S0140-6736(10)60484-9 20609967 PMC2904878

[B6] Grundy SM Cardiovascular and metabolic risk factors: how can we improve outcomes in the high-risk patient? Am J Med 2007 120 9 Suppl 1 S3 S8 discussion S9 17720359 10.1016/j.amjmed.2007.06.005

[B7] Golden SH Wang NY Klag MJ Meoni LA Brancati FL Blood pressure in young adulthood and the risk of type 2 diabetes in middle age Diabetes Care 2003 26 4 1110 1115 10.2337/diacare.26.4.1110 12663582

[B8] Gress TW Nieto FJ Shahar E Wofford MR Brancati FL Hypertension and antihypertensive therapy as risk factors for type 2 diabetes mellitus. Atherosclerosis Risk in Communities Study N Engl J Med 2000 342 13 905 912 10.1056/NEJM200003303421301 10738048

[B9] Wei GS Coady SA Goff DC Jr Brancati FL Levy D Selvin E Vasan RS Fox CS Blood pressure and the risk of developing diabetes in african americans and whites: ARIC, CARDIA, and the framingham heart study Diabetes Care 2011 34 4 873 879 10.2337/dc10-1786 21346180 PMC3064044

[B10] Mullican DR Lorenzo C Haffner SM Is prehypertension a risk factor for the development of type 2 diabetes? Diabetes Care 2009 32 10 1870 1872 10.2337/dc09-0328 19628813 PMC2752916

[B11] Conen D Ridker PM Mora S Buring JE Glynn RJ Blood pressure and risk of developing type 2 diabetes mellitus: the Women’s Health Study Eur Heart J 2007 28 23 2937 2943 10.1093/eurheartj/ehm400 17925342

[B12] Meisinger C Doring A Heier M Blood pressure and risk of type 2 diabetes mellitus in men and women from the general population: the Monitoring Trends and Determinants on Cardiovascular Diseases/Cooperative Health Research in the Region of Augsburg Cohort Study J Hypertens 2008 26 9 1809 1815 10.1097/HJH.0b013e328307c3e9 18698216

[B13] Kramer CK von Muhlen D Barrett-Connor E Mid-life blood pressure levels and the 8-year incidence of type 2 diabetes mellitus: the Rancho Bernardo Study J Hum Hypertens 2010 24 8 519 524 10.1038/jhh.2009.103 20016524 PMC2888977

[B14] Wilhelmsen L Lappas G Rosengren A Risk of coronary events by baseline factors during 28 years follow-up and three periods in a random population sample of men J Intern Med 2004 256 4 298 307 10.1111/j.1365-2796.2004.01372.x 15367172

[B15] Harmsen P Lappas G Rosengren A Wilhelmsen L Long-term risk factors for stroke: twenty-eight years of follow-up of 7457 middle-aged men in Goteborg, Sweden Stroke 2006 37 7 1663 1667 10.1161/01.STR.0000226604.10877.fc 16728686

[B16] Wilhelmsen L Berglund G Elmfeldt D Tibblin G Wedel H Pennert K Vedin A Wilhelmsson C Werko L The multifactor primary prevention trial in Goteborg, Sweden Eur Heart J 1986 7 4 279 288 3720755 10.1093/oxfordjournals.eurheartj.a062065

[B17] Schaufelberger M Rosengren A Heart failure in different occupational classes in Sweden Eur Heart J 2007 28 2 212 218 17185309 10.1093/eurheartj/ehl435

[B18] Wilhelmsen L Berglund G Werko L Prevalence and management of hypertension in a general population sample of Swedish men Prev Med 1973 2 1 57 66 10.1016/0091-7435(73)90008-X 4154438

[B19] World Health Organization-International Society of Hypertension Guidelines for the Management of Hypertension. Guidelines Subcommittee J Hypertens 1999 17 2 151 183 10067786

[B20] McPhillips JB Barrett-Connor E Wingard DL Cardiovascular disease risk factors prior to the diagnosis of impaired glucose tolerance and non-insulin-dependent diabetes mellitus in a community of older adults Am J Epidemiol 1990 131 3 443 453 2301354 10.1093/oxfordjournals.aje.a115519

[B21] Kaplan NM Hypertension curriculum review: lifestyle modifications for prevention and treatment of hypertension J Clin Hypertens (Greenwich) 2004 6 12 716 719 10.1111/j.1524-6175.2004.03610.x 15599122 PMC8109382

[B22] Elliott WJ Meyer PM Incident diabetes in clinical trials of antihypertensive drugs: a network meta-analysis Lancet 2007 369 9557 201 207 10.1016/S0140-6736(07)60108-1 17240286

[B23] Izzo R de Simone G Chinali M Iaccarino G Trimarco V Rozza F Giudice R Trimarco B De Luca N Insufficient control of blood pressure and incident diabetes Diabetes Care 2009 32 5 845 850 10.2337/dc08-1881 19223610 PMC2671117

[B24] Viera AJ Predisease: when does it make sense? Epidemiol Rev 2011 33 1 122 134 10.1093/epirev/mxr002 21624963

[B25] Feihl F Liaudet L Waeber B Levy BI Hypertension: a disease of the microcirculation? Hypertension 2006 48 6 1012 1017 10.1161/01.HYP.0000249510.20326.72 17060505

[B26] Nguyen TT Wang JJ Islam FM Mitchell P Tapp RJ Zimmet PZ Simpson R Shaw J Wong TY Retinal arteriolar narrowing predicts incidence of diabetes: the Australian Diabetes, Obesity and Lifestyle (AusDiab) Study Diabetes 2008 57 3 536 539 10.2337/db07-1376 18086902

[B27] Serne EH Stehouwer CD ter Maaten JC ter Wee PM Rauwerda JA Donker AJ Gans RO Microvascular function relates to insulin sensitivity and blood pressure in normal subjects Circulation 1999 99 7 896 902 10.1161/01.CIR.99.7.896 10027812

[B28] Levy BI Schiffrin EL Mourad JJ Agostini D Vicaut E Safar ME Struijker-Boudier HA Impaired tissue perfusion: a pathology common to hypertension, obesity, and diabetes mellitus Circulation 2008 118 9 968 976 10.1161/CIRCULATIONAHA.107.763730 18725503

[B29] Houben AJ Eringa EC Jonk AM Serne EH Smulders YM Stehouwer CD Perivascular Fat and the Microcirculation: Relevance to Insulin Resistance, Diabetes, and Cardiovascular Disease Curr Cardiovasc Risk Rep 2012 6 1 80 90 10.1007/s12170-011-0214-0 22247785 PMC3251783

[B30] Thuillez C Richard V Targeting endothelial dysfunction in hypertensive subjects J Hum Hypertens 2005 19 Suppl 1 S21 S25 16075029 10.1038/sj.jhh.1001889

[B31] Meigs JB O’Donnell CJ Tofler GH Benjamin EJ Fox CS Lipinska I Nathan DM Sullivan LM D’Agostino RB Wilson PW Hemostatic markers of endothelial dysfunction and risk of incident type 2 diabetes: the Framingham Offspring Study Diabetes 2006 55 2 530 537 10.2337/diabetes.55.02.06.db05-1041 16443791

[B32] Sesso HD Buring JE Rifai N Blake GJ Gaziano JM Ridker PM C-reactive protein and the risk of developing hypertension JAMA 2003 290 22 2945 2951 10.1001/jama.290.22.2945 14665655

[B33] Hu FB Meigs JB Li TY Rifai N Manson JE Inflammatory markers and risk of developing type 2 diabetes in women Diabetes 2004 53 3 693 700 10.2337/diabetes.53.3.693 14988254

[B34] Salas-Salvado J Bullo M Babio N Martinez-Gonzalez MA Ibarrola-Jurado N Basora J Estruch R Covas MI Corella D Aros F Reduction in the incidence of type 2 diabetes with the Mediterranean diet: results of the PREDIMED-Reus nutrition intervention randomized trial Diabetes Care 2011 34 1 14 19 10.2337/dc10-1288 20929998 PMC3005482

