# Supplementary material for: High-normal blood pressure and long-term risk of type 2 diabetes: 35-year prospective population based cohort study of men
Source: BMC Cardiovasc Disord. 2012 Oct 15;12:89. doi: 10.1186/1471-2261-12-89 (PMC3509395; doi:10.1186/1471-2261-12-89)
Supplement: Additional file 1 — Table S3. a. Hazard ratio for diabetes by systolic blood pressure (SBP) categories in different BMI categories. b; Hazard ratio for diabetes by systolic blood pressure (SBP) categories in different smoking categories. [file 1471-2261-12-89-S1.docx]

**Table 3a**; Hazard ratio for diabetes by systolic blood pressure (SBP) categories in different BMI categories.

| **SBP categories by different BMI categories** | **Number at risk** | **Diabetes cases** | **Person**  **years** | **Diabetes cases**  **per 1 00 000**  **person**  **years** | **Age adjusted hazard ratios**  **(95% CI)** | **Age and multivariable adjusted* hazard ratios**  **(95% CI)** |
| --- | --- | --- | --- | --- | --- | --- |
| **BMI < 25** |  |  |  |  |  |  |
| < 130 | 768 | 38 | 21924 | 173 | ref | ref |
| 130 – 139 | 649 | 53 | 17549 | 302 | 1.87 (1.23-2.84) | 1.93 (1.27-2.94) |
| 140 – 159 | 1174 | 87 | 31525 | 276 | 1.71 (1.17-2.51) | 1.73 (1.17-2.55) |
| ≥ 160 | 782 | 64 | 18617 | 344 | 2.40 (1.60-3.59) | 2.38 (1.57-3.61) |
| Increase per  10 mm Hg |  |  |  |  | 1.12 (1.05-1.18) | 1.10 (1.05-1.18) |
| **BMI 25-30** |  |  |  |  |  |  |
| < 130 | 468 | 59 | 13275 | 444 | ref | ref |
| 130 – 139 | 592 | 88 | 16091 | 547 | 1.28 (0.92-1.78) | 1.23 (0.89-1.72) |
| 140 – 159 | 1233 | 185 | 33096 | 559 | 1.31 (0.97-1.75) | 1.23 (0.91-1.65) |
| ≥ 160 | 1068 | 197 | 26927 | 732 | 1.85 (1.38-2.47) | 1.56 (1.15-2.10) |
| Increase per  10 mm Hg |  |  |  |  | 1.10 (1.06-1.15) | 1.07 (1.03-1.12) |
| **BMI 30+** |  |  |  |  |  |  |
| < 130 | 43 | 12 | 1094 | 1 097 | ref | ref |
| 130 – 139 | 73 | 18 | 1885 | 955 | 0.83 (0.40-1.73) | 1.06 (0.49-2.31) |
| 140 – 159 | 214 | 58 | 5194 | 1 117 | 0.98 (0.53-1.82) | 1.12 (0.57-2.20) |
| ≥ 160 | 267 | 97 | 6152 | 1 577 | 1.50 (0.82-2.74) | 1.68 (0.86-3.27) |
| Increase per  10 mm Hg |  |  |  |  | 1.12 (1.05-1.20) | 1.10 (1.03-1.18) |

*Multivariable model was adjusted for age, cholesterol, antihypertensive treatment, smoking, physical activity and social class.

**Table 3b**; Hazard ratio for diabetes by systolic blood pressure (SBP) categories in different smoking categories.

| **SBP categories by different smoking categories** | **Number at risk** | **Diabetes cases** | **Person**  **years** | **Diabetes cases**  **per 1 00 000**  **person years** | **Age adjusted hazard ratios**  **(95% CI)** | **Age and multivariable adjusted* hazard ratios**  **(95% CI)** |
| --- | --- | --- | --- | --- | --- | --- |
| **Non-smokers** |  |  |  |  |  |  |
| < 130 | 347 | 34 | 10655 | 319 | ref | ref |
| 130 – 139 | 355 | 43 | 10491 | 410 | 1.36 (0.86-2.13) | 1.21 (0.77-1.90) |
| 140 – 159 | 775 | 92 | 22595 | 407 | 1.32 (0.89-1.96) | 1.04 (0.69-1.54) |
| ≥ 160 | 675 | 124 | 18120 | 684 | 2.43 (1.66-3.55) | 1.51 (1.01-2.27) |
| Increase per  10 mm Hg |  |  |  |  | 1.16 (1.10-1.21) | 1.08 (1.03-1.14) |
| **Former smokers** |  |  |  |  |  |  |
| <130 | 242 | 17 | 7182 | 237 | ref | ref |
| 130-139 | 273 | 40 | 7843 | 510 | 2.26 (1.28-3.98) | 1.89 (1.07-3.34) |
| 140-159 | 555 | 80 | 15619 | 512 | 2.32 (1.38-3.92) | 1.73 (1.02-2.95) |
| ≥160 | 423 | 82 | 11097 | 739 | 3.69 (2.18-6.23) | 2.43 (1.41-4.18) |
| Increase per  10 mm Hg |  |  |  |  | 1.15 (1.08-1.22) | 1.10 (1.04-1.18) |
| **Current smokers** |  |  |  |  |  |  |
| < 130 | 686 | 56 | 18348 | 305 | ref | Ref |
| 130 – 139 | 681 | 76 | 17080 | 445 | 1.54 (1.09-2.18) | 1.46 (1.03-2.07) |
| 140 – 159 | 1279 | 156 | 31338 | 498 | 1.75 (1.29-2.38) | 1.50 (1.10-2.04) |
| ≥ 160 | 1014 | 151 | 22328 | 676 | 2.63 (1.93-3.58) | 2.01 (1.46-2.77) |
| Increase per  10 mm Hg |  |  |  |  | 1.17 (1.13-1.22) | 1.12 (1.06-1.16) |

*Multivariable model was adjusted for age, BMI, cholesterol, antihypertensive treatment, physical activity and social class.
